# Combination of BMI1 and MAPK/ERK inhibitors is effective in medulloblastoma

**DOI:** 10.1093/neuonc/noac052

**Published:** 2022-02-25

**Authors:** Sara Badodi, Nicola Pomella, Yau Mun Lim, Sebastian Brandner, Gillian Morrison, Steven M Pollard, Xinyu Zhang, Nicolae Radu Zabet, Silvia Marino

**Affiliations:** Blizard Institute, Barts and The London School of Medicine and Dentistry, Queen Mary University of London, London, UK; Blizard Institute, Barts and The London School of Medicine and Dentistry, Queen Mary University of London, London, UK; UCL Queen Square Institute of Neurology and The National Hospital for Neurology and Neurosurgery, University College London Hospitals NHS Foundation Trust, London, UK; UCL Queen Square Institute of Neurology and The National Hospital for Neurology and Neurosurgery, University College London Hospitals NHS Foundation Trust, London, UK; Centre for Regenerative Medicine & Cancer Research UK Edinburgh Centre, The University of Edinburgh, Edinburgh, UK; Centre for Regenerative Medicine & Cancer Research UK Edinburgh Centre, The University of Edinburgh, Edinburgh, UK; Blizard Institute, Barts and The London School of Medicine and Dentistry, Queen Mary University of London, London, UK; Blizard Institute, Barts and The London School of Medicine and Dentistry, Queen Mary University of London, London, UK; Blizard Institute, Barts and The London School of Medicine and Dentistry, Queen Mary University of London, London, UK

**Keywords:** BMI1, CHD7, epigenetic, medulloblastoma, MAPK/ERK

## Abstract

**Background:**

Epigenetic changes play a key role in the pathogenesis of medulloblastoma (MB), the most common malignant pediatric brain tumor.

**Methods:**

We explore the therapeutic potential of BMI1 and MAPK/ERK inhibition in BMI1^High^;CHD7^Low^ MB cells and in a preclinical xenograft model.

**Results:**

We identify a synergistic vulnerability of BMI1^High^;CHD7^Low^ MB cells to a combination treatment with BMI1 and MAPK/ERK inhibitors. Mechanistically, CHD7-dependent binding of BMI1 to MAPK-regulated genes underpins the CHD7-BMI1-MAPK regulatory axis responsible of the antitumour effect of the inhibitors *in vitro* and in a preclinical mouse model. Increased ERK1 and ERK2 phosphorylation activity is found in BMI1^High^;CHD7^Low^ G4 MB patients, raising the possibility that they could be amenable to a similar therapy.

**Conclusions:**

The molecular dissection of the CHD7-BMI1-MAPK regulatory axis in BMI1^High^;CHD7^Low^ MB identifies this signature as a proxy to predict MAPK functional activation, which can be effectively drugged in preclinical models, and paves the way for further exploration of combined BMI1 and MAPK targeting in G4 MB patients.

**Key Points**

- Combination of BMI1 and MAPK/ERK inhibitors is effective in medulloblastoma.
- BMI1^High^;CHD7^Low^ signature defines G4 MB patients with an enhanced ERK1/ERK2 activity.
- BMI1^High^;CHD7^Low^ is a biomarker that can predict response to BMI1 and MAPK/ERK inhibitors.

**Importance of the Study**

Medulloblastoma (MB) is the most common pediatric malignant brain tumor. Recent advances in MB classification have not yet translated into a biologically tailored therapy, hence the development of novel approaches exploiting the acquired biological understanding is now a priority.

Here we demonstrate that the BMI1^High^;CHD7^Low^ molecular signature defines G4 MB patients with an enhanced ERK1/ERK2 phosphorylation activity that could benefit from a combination treatment with BMI1 and MAPK/ERK inhibitors, currently singularly used in clinical trials for other brain tumors. Importantly, transcriptomic data are widely used in MB diagnosis, thus it is envisaged that the BMI1^High^;CHD7^Low^ signature, which functions as a biomarker predicting response to BMI1 and MAPK/ERK inhibitors, could be readily implemented in clinical practice.

In summary, we found an actionable vulnerability of a molecularly defined group of MB patients, paving the way for the design of much-needed molecularly matched therapies.

Medulloblastoma (MB) is the most common malignant pediatric brain tumor. A combination of genomic, epigenomic, and transcriptomic studies have informed a consensus molecular classification of MB into four distinct molecular subgroups (WNT, SHH, Group 3, and Group 4), each of them further subdivided into subtypes with distinct prognosis and responses to therapy.^1,2^ However, these significant advances in the classification of MB have so far not translated into a biologically tailored therapy for patients, which continue to be treated with surgical resection followed by chemotherapy/radiotherapy irrespective of the molecular features of their tumor. Although this multi-modal treatment results in a favorable overall survival rate, recurrence, and metastasis are still incurable, making MB the leading cause of childhood mortality due to cancer. Moreover, long-term survivors face significant treatment-related morbidity and reduced quality of life, mainly due to neurotoxicity, cognitive dysfunction, and neuropsychological deficits.^3^ Risk stratification of patients based on molecular subtyping and targeted therapies are essential to reduce secondary effects and improve overall prognosis and quality of life.

Epigenetic mechanisms play a key role during tumor formation and progression, and alterations in epigenetic modifiers have been detected in many tumor types.^4,5^ Genome-wide screens of MB^6–8^ revealed essential roles of histone modifiers, chromatin remodeling factors, and over-expression of Polycomb-group (PcG) proteins^9–11^ particularly in Group 4 (G4) subtype, indicating that epigenetic changes can represent driver events in MB. The PcG protein BMI1 is involved in regulation of fate determination and proliferation of normal stem cells, including neural stem cells (NSC).^11–13^ Deregulated BMI1 function sustains tumor maintenance and progression; indeed, its expression is frequently upregulated in a variety of cancers, including brain tumors such as diffuse intrinsic pontine glioma (DIPG),^14^ and MB.^9,10^ In particular, BMI1 is highly expressed in G4 MB.^9,15,16^ We previously showed that BMI1 and the ATP-dependent chromatin remodeler CHD7 (Chromodomain-Helicase-DNA binding protein 7) collaborate in MB pathogenesis and we described a BMI1^High^;CHD7^Low^ molecular signature in G4 MB patients with poor prognosis where it sustains tumor growth through regulation of proliferation and metabolic adaptation.^9,16^

Many molecular pathways sustaining tumor growth converge onto the mitogen-activated protein kinase (MAPK) extracellular regulated kinase (ERK) signaling. In particular, ERK1/2 are phosphorylated and translocated to the nucleus where they regulate the function of different transcription factors, including ETS domain-containing proteins ELK and ELF.^17^ Aberrant induction of MAPK/ERK signaling can sustain different aspects of tumor biology, including proliferation, invasion, and metastasis, but also therapy resistance.^18^ Multiple events driving uncontrolled activation of MAPK/ERK and affecting different levels of the cascade were described in a broad range of cancers including MB.^19,20^ Notably, ERBB4-SRC signaling has been identified as a hallmark of G4 MB subtype, which implies activation of downstream effectors such as MAPK/ERK signaling.^21^

MAPK/ERK signaling is regulated by multiple epigenetic mechanisms, targeting the different steps of the activation process. microRNAs can regulate various proteins involved in the signaling activation,^22^ while DNA methylation and histone modifiers can affect MAPK/ERK signaling via epigenetic silencing of DUSP phosphatases, which are negative regulators of the pathways.^9,23,24^ These regulatory interactions between epigenetic modifiers and MAPK/ERK pathway raise the possibility that they may represent vulnerabilities that could be targeted by combined therapeutic approaches.

Here we explore the therapeutic potential of BMI1 and MAPK/ERK inhibition in MB cells and a preclinical xenograft model. We elucidate the molecular mechanism underlying the CHD7-BMI1-MAPK regulatory axis to inform future translational applications in BMI1^High^;CHD7^Low^ MB patients.

## Methods

### Cell Culture Conditions

ICb1299 and CHLA-01-Med MB cells were cultured as previously described.^16,25,26^ Human fetal NSC lines were obtained from the Cancer Research UK-funded Glioma Cellular Genetics Resource (www.gcgr.org.uk) and cultured as previously described.^16^

### Cell Viability, Growth, and Drug Interaction Assays

Cells were treated with PTC-209 (Tocris) and PD325901 (Tocris) both dissolved in DMSO (Sigma) at the indicated concentrations. At specific time points and after appropriate treatment, cells were harvested, and the number of viable cells was counted with a hemocytometer and Trypan Blue staining or with CyQUANT Direct Red Cell Proliferation Assay Kit (ThermoFisher Scientific). Synergy/antagonist effect of the combined treatment was analyzed as previously described.^16^ See Supplementary Methods.

### Western Blot Analysis

Western blot analysis on total or nuclear extracts was performed as previously described.^16,27^ See Supplementary Methods.

### RNA-Seq, ChIP-Seq, and Bioinformatic Analysis

RNA and ChIP sequencing (peak annotation and motif enrichment) analysis were performed as previously described.^16^ See Supplementary Methods for details about specific bioinformatics analysis.

### Animal Experiments and Ethics Statement

All procedures were performed in accordance with licenses held under the UK Animals (Scientific Procedures) Act 1986 and later modifications and conforming to all relevant guidelines and regulations. Required sample sizes were calculated by an a priori power analysis and mice were assigned randomly to the different groups of treatment. About 1 x 10^5^ MB cells were injected into the right cerebellar hemisphere of P5 mice. After 3 weeks from injection, mice were treated with PTC-209 60mg/kg + PD325901 25mg/kg diluted in 5% DMSO (Sigma)/ 10% Kolliphor (Sigma)/ 85% PBS without Ca^+^ and Mg^+^ (Gibco). Control mice were i.p. injected with 5% DMSO/ 10% Kolliphor/ 85% PBS solution, as vehicle.

### Data Analysis and Statistical Methods

Number of experiments (*n*) used for all the statistical tests is specified in each figure legends. All quantitative data are presented as mean ± standard error of the mean (SEM) of at least three experiments, unless otherwise specified. The statistical significance was determined by two-tailed unpaired Student’s t-test, one-way ANOVA followed by Tukey’s multiple comparison test, or two-way ANOVA followed by Dunnet’s multiple comparison test. Survival of xenografted mice was estimated with Kaplan-Meier survival analysis and significance was determined with Log-rank (Mantel-Cox) test. Statistical analysis was performed using GraphPad Prism software and statistical significance is represented as: *P < .05, **P < .01, ***P < .001 or ****P < .0001.

## Results

### CHD7 Silencing Renders MB Cells More Susceptible to BMI1 Inhibitor Treatment

**Fig. 1:**
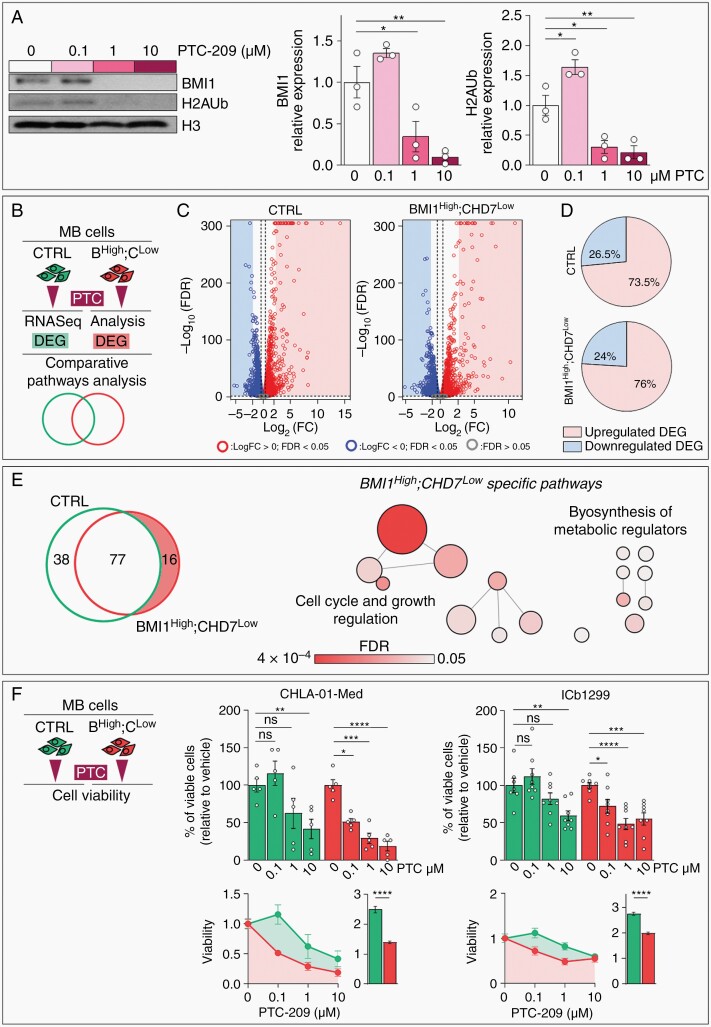
BMI1^High^;CHD7^Low^ cells are more responsive to BMI1-mediated cell growth inhibition. A. Western blot and quantification of BMI1 and mono-ubiquityl-Histone H2A (H2AUb) levels after 24h treatment with increasing concentrations of PTC-209. HISTONE H3 was used to normalize protein loading. *n* = 3 independent biological experiments, one-way ANOVA. B. Schematic representation of the comparative analysis between control (CTRL) and BMI1^High^;CHD7^Low^ MB cell lines performed to identify differentially expressed genes (DEG) and common or specific deregulated pathways upon 24h of 1µM PTC treatment. C. Volcano plots of DEG between PTC- and vehicle-treated CTRL or BMI1^High^;CHD7^Low^ ICb1299 MB cells. Red and blue dots represent genes with Log_2_FC >0 or <0 and FDR<0.05 respectively, genes not significantly regulated are represented in gray. D. Pie charts representing percentages of significantly upregulated or downregulated DEG respectively with Log_2_FC>2 or Log_2_FC<-2. E. Venn diagram of canonical pathways enriched for in PTC-mediated DEG in CTRL or BMI1^High^;CHD7^Low^. Highlighted BMI1^High^;CHD7^Low^-specific pathways (left panel). Bubbles plot showing BMI1^High^;CHD7^Low^-specific canonical pathways. Bubbles are colored based on FDR values and size is proportional to number of genes of specific pathways (right panel). F. Cell viability assays of CTRL (green) or BMI1^High^;CHD7^Low^ (B^High^;C^Low^, red) CHLA-01-Med and ICb1299 MB cells upon treatment with increasing concentrations of PTC-209. Histograms represent percentages of viable cells relative to vehicle-treated cells (top). Measurement of Area-under-Curve (AUC) and histograms representing mean AUC were used to compare the overall response to treatment (bottom). *n* = 5 independent biological experiments for CHLA-01-Med or *n* = 8 for ICb1299, two-way ANOVA. All graphs report mean ± SEM.

To test the therapeutic potential of BMI1 targeting in MB we used PTC-209, a BMI1 small molecule inhibitor. As previously described in other cancer models,^28–30^ BMI1 protein level is reduced in MB cells treated with increasing concentrations of PTC-209 (Figure 1A). Reduction of global mono-ubiquitination of histone H2A (H2AUb) in treated MB cells (Figure 1A) is also observed, in keeping with BMI1 function as a core protein of the polycomb repressive complex 1 (PRC1) responsible for epigenetic repression through histone ubiquitination.^31^ We previously described a BMI1^High^;CHD7^Low^ signature in G4 MB, including its relevance in the pathogenesis of these tumors.^9^ Hence, we set out to investigate whether any effect of a BMI1 pharmacological inhibition would be CHD7-dependent. We modeled the BMI1^High^;CHD7^Low^ signature in MB cells overexpressing BMI1 (compared to normal cerebellum), by CHD7 silencing, as previously described (Supplementary Figure S1A).^9^ We analyzed the transcriptome of MB cells upon a short-term (24 h) PTC-209 treatment, and compared differentially expressed genes (DEG) and related canonical pathways in control vs BMI1^High^;CHD7^Low^ cells (Figure 1B). We identified 8457 (4129 up- and 4328 downregulated) and 7794 (3902 up- and 3892 downregulated) significant DEG respectively in control and BMI1^High^;CHD7^Low^ MB cells (Figure 1C). Focusing the analysis on the most deregulated genes (i.e. with a log fold change of a magnitude equal or higher then 2), we show that BMI1 inhibition mainly induced de-repression, hence upregulation, of genes (Figure 1D), confirming that BMI1 pharmaceutical inhibition impacts its canonical epigenetic role leading to transcriptional repression. Importantly, enrichment for pathways involved in cell cycle and cell proliferation and in the biosynthesis of molecules responsible for metabolic regulation was found when BMI1^High^;CHD7^Low^-specific pathways were considered, suggesting that CHD7 mediates the impact of BMI1 pharmacological inhibition on pathways supporting cell growth and viability (Figure 1E and Supplementary Figure S1B). Consistently, two independent BMI1^High^;CHD7^Low^ MB cell lines were found to be more responsive to PTC-mediated cell growth inhibition upon treatment with increasing concentrations of PTC-209 (Figure 1F). On the contrary, control cells were affected only by the highest PTC dose used (Figure 1F). In line with this observation, modeling of the signature induced an over 10-fold decrease of PTC IC50 in MB cells (0.08 µM compared to 0.95–1.05 µM in control cells, Supplementary Figure S1C). Interestingly, PTC-209 does not affect viability of cerebellar-derived human neural stem cells (hNSC) irrespective of the presence of BMI1^High^;CHD7^Low^ signature (Supplementary Figure S1D,E), suggesting a negligible side effect of the treatment, as described in the context of other non-neoplastic cells.^30,32^ Remarkably, CHD7 over-expression blunts PTC-sensitivity in BMI1^High^;CHD7^Low^ MB cells irrespective of drug dosage (Supplementary Figure S1F,G).

Our data show that BMI1^High^;CHD7^Low^ signature sensitizes MB cells to impaired viability mediated by BMI1 pharmacological inhibition.

### Impaired Cell Growth in BMI1^High^;CHD7^Low^ MB Cells Upon MAPK/ERK Inhibition

**Fig. 2:**
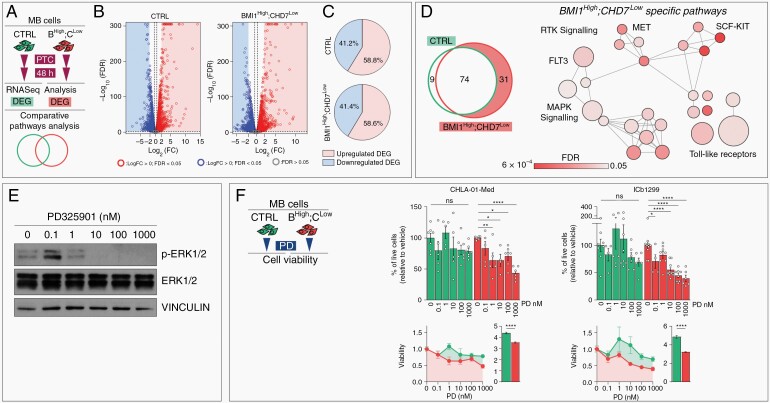
BMI1^High^;CHD7^Low^ signature renders MB cells more responsive to MAPK inhibition. A. Schematic representation of the comparative analysis between control (CTRL) and BMI1^High^;CHD7^Low^ (B^High^;C^Low^) MB cell lines performed to identify differentially expressed genes (DEG) and shared or specific deregulated pathways upon 48h of 1µM PTC-209 (PTC) treatment. B–C. (B) Volcano plots of DEG between 48h PTC- and vehicle-treated in CTRL or BMI1^High^;CHD7^Low^ ICb1299 MB cells. Red and blue dots represent genes with Log_2_FC >0 or <0 and FDR < 0.05 respectively, genes not significantly regulated are represented in gray. (C) Pie charts representing percentages of significantly upregulated or downregulated DEG respectively with Log_2_FC>2 or Log_2_FC<-2. D. Venn diagram of canonical pathways enriched for in 48h PTC-mediated DEG in CTRL or BMI1^High^;CHD7^Low^. Highlighted BMI1^High^;CHD7^Low^-specific pathways (left panel). Bubbles plot showing BMI1^High^;CHD7^Low^-specific Reactome pathways. Bubbles are colored based on FDR values and size is proportional to number of genes of specific pathways (right panel). E. Representative images of Western blot analysis of phosphorylated/total ERK1/2 in ICb1299 MB cells treated for 24h with increasing concentrations of PD329501. VINCULIN was used to normalize protein loading. F. Cell viability assays of CTRL (green) or BMI1^High^;CHD7^Low^ (red) CHLA-01-Med and ICb1299 MB cells upon treatment with increasing concentrations of PD329501 (PD). Histograms represent percentages of viable cells relative to vehicle-treated cells (top). Measurement of Area-under-Curve (AUC) and histograms representing mean AUC were used to compare the overall response to treatment (bottom). *n* = 5–7 independent biological experiments, two-way ANOVA. All graphs report mean ± SEM.

Suppression of epigenetic regulators is linked to acquired therapy resistance.^33^ We investigated indirect transcriptome modulation mediated by BMI1 inhibition upon 48h treatment with PTC-209 in control vs BMI1^High^;CHD7^Low^ MB cells (Figure 2A). We identified 9057 (4486 up- and 4517 downregulated) and 8193 (4100 up- and 4093 downregulated) DEG in control or upon signature modeling respectively (Figure 2B). As for early PTC-responder genes, we found that deregulated genes were predominantly upregulated (Figure 2C), in keeping with BMI1-mediated epigenetic repression (Figure 1D), but the genes identified were not identical, indicating a further modulation of gene expression not directly linked to BMI1 inhibition.

Comparative analysis identified enriched canonical pathways mainly related to Receptor Tyrosine Kinase (RTK), including MET, FLT3, and SCF-KIT as well as MAPK/ERK signaling (Figure 2D), specifically in DEG of PTC-treated BMI1^High^;CHD7^Low^ MB cells, in keeping with the previously reported regulation of DUSP4 expression (dual-specificity phosphatase 4), a phosphatase which dephosphorylates residues of MAPK substrates, in these cells. Therefore, we treated two independent MB cell lines with PD325901 (hereafter named PD), a MEK inhibitor that blocks ERK1/2 phosphorylation (Figure 2E). We found that BMI1^High^;CHD7^Low^ cells are more responsive to impaired viability induced by MAPK/ERK inhibition compared to control cells (Figure 2F), as demonstrated also by lower IC50 upon modeling of the signature (Supplementary Figure S2A). CHD7 over-expression blunts PD-sensitivity in BMI1^High^;CHD7^Low^ MB cells irrespective of drug dosage (Supplementary Figure S2C). hNSC viability is not affected by PD treatment irrespective of the presence of BMI1^High^;CHD7^Low^ signature (Supplementary Figure S2B).

In conclusion, our data show that gene expression modulated by BMI1 affects RTK and MAPK/ERK signaling upon BMI1^High^;CHD7^Low^ modeling, a condition rendering these cells sensitive to MAPK/ERK inhibition.

### A CHD7-BMI1-MAPK Regulatory Axis in BMI1^High^;CHD7^Low^ MB Cells

**Fig. 3:**
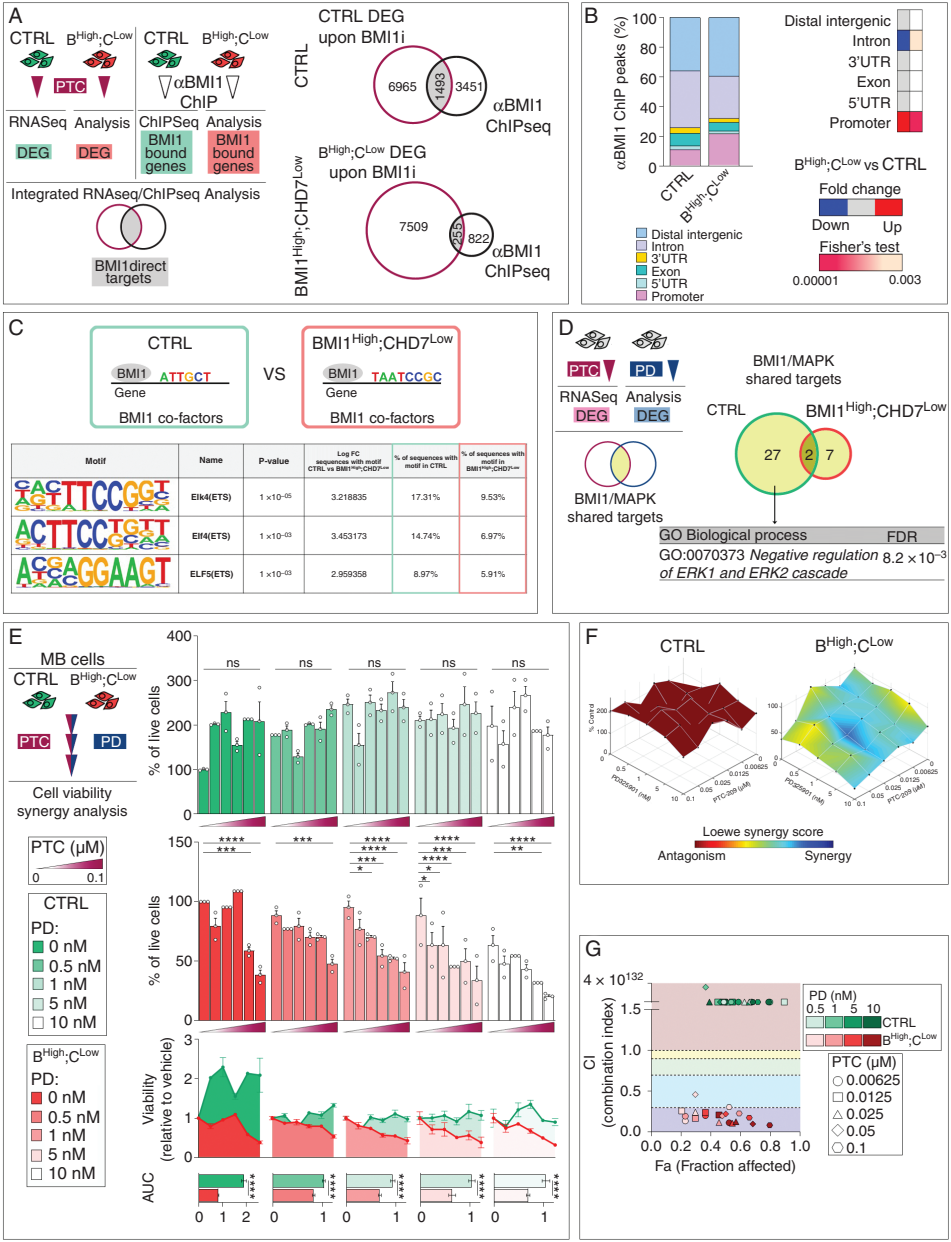
A synergistic activity of BMI1 and MAPK/ERK inhibitors in BMI1^High^;CHD7^Low^ MB cells. A. Schematic representation of the integrative analysis between RNA-Seq data upon PTC treatment, and αBMI1ChIP-Seq of control (CTRL) and BMI1^High^;CHD7^Low^ (B^High^;C^Low^) CHLA-01-Med and ICb1299 MB cells performed to identify BMI1 direct targets (left). Venn diagrams representing integrated RNA-Seq and Chip-Seq analysis identifying BMI1 targets (right). B. Distribution of genomic annotations of ChIP-Seq peaks of BMI1 targets identified in A (left). Heatmap represents Fisher’s exact test statistical analysis of fold changes in percentages of BMI1 ChIP-peaks upon modeling of BMI1^High^;CHD7^Low^ (BMI1^High^;CHD7^Low^ vs CTRL). C. Motif analysis of BMI1-bound peaks in CTRL compared to BMI1^High^;CHD7^Low^ CHLA-01-Med and ICb1299 MB cells. D. Schematic representation of the comparative analysis between CTRL and BMI1^High^;CHD7^Low^ CHLA-01-Med and ICb1299 MB cell lines performed to identify common differentially expressed genes (DEG) upon PTC or PD treatment (BMI1/MAPK shared targets, represented in Supplementary Figure S3C,D) (left). Venn diagram showing overlap of BMI1/MAPK shared targets between CTRL and BMI1^High^;CHD7^Low^ MB cells and GO Analysis of the 27 CTRL-specific target genes (right). E. Cell viability assays of CTRL (green) or BMI1^High^;CHD7^Low^ (red) CHLA-01-Med MB cells upon 72 hours of combination treatment with increasing concentrations of PTC and PD. Histograms represent percentages of viable cells relative to vehicle-treated cells. Measurement of Area-under-Curve (AUC) and histograms representing mean AUC were used to compare the overall response to treatment. *n* = 3 independent biological experiments, two-way ANOVA. F. Surface plots of Loewe Synergy Scores obtained after combination treatment in CTRL or BMI1^High^;CHD7^Low^ cells. Individual values used to obtain surface plots are shown in Figure S3F. *n* = 3 biological independent experiments. G. Combination Index (CI)-effect (Fa, Fraction affected) plot for CTRL or BMI1^High^;CHD7^Low^ cells treated with the indicated combination of PTC (different symbols) and PD (different shades of colors). Colored area of the plot delineate CI value ranges representing: CI<0.3 = strong synergy, CI:0.3–0.7 = moderate synergy, CI:0.7–0.9 = mild synergy, CI:0.9–1=additive, CI>1 = antagonism. All graphs report mean ± SEM.

To establish the mechanism of CHD7-dependent BMI1-mediated overactivation of MAPK/ERK pathway, we set out to identify direct targets of BMI1 by integrating the RNA-Seq analysis upon 24h PTC treatment with ChIP-Seq data describing the genome-wide distribution of BMI1 in two independent BMI1^High^;CHD7^Low^ MB cell lines.^16^ We retrieved BMI1 direct targets, defined as genes with a significant deregulated expression upon inhibition of BMI1 activity (PTC-responding genes) and displaying a BMI1 ChIP-Seq peak (Figure 3A). Annotation of αBMI1 peaks on the identified direct targets revealed a redistribution of BMI1 across the genome upon CHD7 silencing, with occupancy significantly increased in promoters and decreased in intronic regions (10.6 vs 21.2% on promoters and 38.3 vs 28.6% on intron respectively in control vs BMI1^High^;CHD7^Low^, Fisher’s exact test: *P* < .0001 and *P* = .003, Figure 3B). Interestingly, CHD7-mediated change in BMI1 occupancy at promoter was specifically linked to genes that were derepressed upon PTC treatment (Supplementary Figure S3A,B), suggesting that CHD7 contributes to the canonical epigenetic transcriptional repression mediated by BMI1.

Given the relevance of CHD7 expression for BMI1 binding to promoters, we set out to investigate BMI1 co-factors at these promoters and whether this phenomenon could explain the convergence of BMI1 and CHD7 on MAPK/ERK signaling. Motif analysis of BMI1-bound promoters identified enriched motifs for ETS transcription factors, known MAPK substrates,^17^ including ELK4, ELF4, and ELF5, in control cells compared to CHD7-silenced cells (Figure 3C), indicating that BMI1 binds MAPK-regulated genes specifically when CHD7 is expressed. Therefore, to identify possible BMI1 and MAPK/ERK shared targets, we interrogated the transcriptome of PTC- (BMI1-responding genes) or PD-treated (MAPK-responding genes) MB cells to determine common DEG, whose expression is changed in the same direction upon BMI1 or MAPK inhibition in control or BMI1^High^;CHD7^Low^ MB cells (Figure 3D and Supplementary Figure S3C,D). Gene Ontology (GO) Biological Process analysis revealed that the 27 control-specific BMI1/MAPK shared targets are associated with the *Negative regulation of ERK1 and ERK2 cascade* (Figure 3D), further demonstrating the presence of a CHD7-BMI1-MAPK regulatory axis in MB cells. These data add a novel regulatory layer to the previously described BMI1/CHD7 convergence on DUSP4 regulation,^9^ which was further substantiated in our current analysis where we identified DUSP6 as uniquely bound by BMI1 in control cells expressing CHD7 (Supplementary Figure S3E).

Overall, our results demonstrate a critical role of the CHD7-BMI1-MAPK regulatory axis in viability control of BMI1^High^;CHD7^Low^ MB cells.

### BMI1 and MAPK/ERK Combination Treatment is Antitumourigenic In Vitro and Overcomes Acquired Resistance to Single Drug Therapy

Following on from the evidence of a CHD7-BMI1-MAPK regulatory axis in MB cells, we set out to assess whether BMI1 and MAPK inhibitors could be used in combination to potentiate their anti-tumor activity. We performed drug interaction assays treating control or BMI1^High^;CHD7^Low^ MB cells with increasing combination of PTC and PD doses (Figure 3E). First, we found that BMI1 and MAPK/ERK inhibitors cooperate in reducing cell growth only in MB cells with the signature (Figure 3E). Notably, this was achieved with lower concentrations of both PTC and PD as compared to doses used for single drug assays (Figures 1F and 2F). Next, we assessed PTC:PD interaction by dose-effect analysis based on the Loewe additivity model and showed that PTC and PD synergize uniquely in BMI1^High^;CHD7^Low^ cells (Figure 3F and Supplementary Figure S3F). Finally, combination index values confirmed that PTC and PD have a synergic interaction (CI<1) only in BMI1^High^;CHD7^Low^ cells (Figure 3G and Supplementary Figure S3G) and that the majority of the combination doses used show a strong synergy (CI<0.3, Figure 3G).

**Fig. 4:**
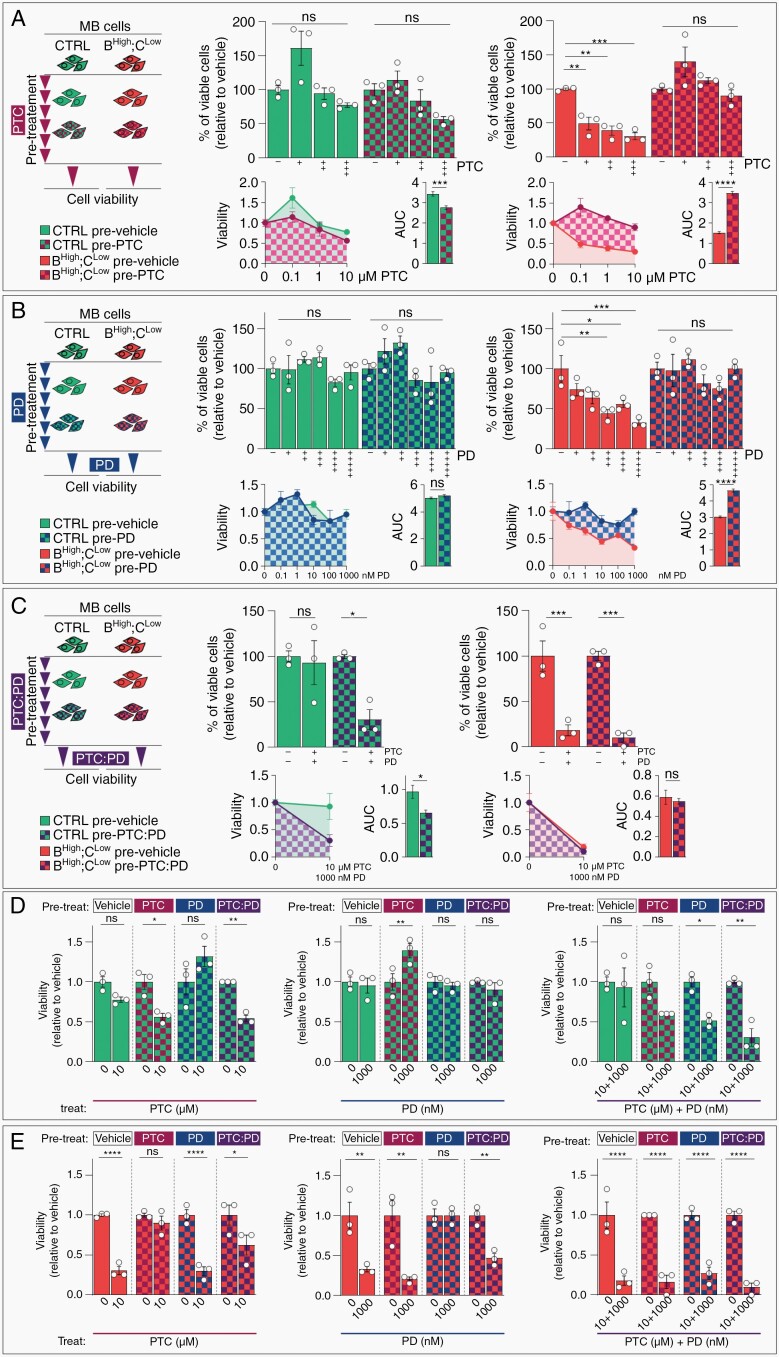
Combined BMI1 and MAPK inhibition overcomes acquired resistance to single drug therapy in BMI1^High^;CHD7^Low^ MB cells. A–C. Cell viability assays of control (CTRL, green) or BMI1^High^;CHD7^Low^ (red) CHLA-01-Med MB cells pre-treated for 6 days with PTC 0.1μM (A), PD 10nM (B) or both (C) and upon treatment with increasing concentrations of PTC-209 (PTC,A), PD329501 (PD,B) or both (PTC:PD,C). Histograms represent percentages of viable cells relative to vehicle-treated (DMSO) cells (top). Measurement of Area-under-Curve (AUC) and histograms representing mean AUC were used to compare the overall response to treatment (bottom). *n* = 3 independent biological experiments, two-way ANOVA. D–E. Cell viability assays of CTRL (D) or BMI1^High^;CHD7^Low^ (E) CHLA-01-Med MB cells pre-treated with PTC, PD or PTC:PD and upon treatment with PTC, PD or PTC:PD. *n* = 3 independent biological experiments, two-way ANOVA. All graphs report mean ± SEM.

To explore whether acquired resistance could compromise drug response, we set up a continuous exposure model where MB cells are pretreated with PTC, PD, or PTC:PD. We found that pre-treatments with PTC (Figure 4A) or PD (Figure 4B) led to single-agent sensitivity loss in BMI1^High^;CHD7^Low^ cells. On the contrary, continuous PTC:PD exposure did not elicit acquired resistance to this combinatorial treatment (Figure 4C). Interestingly, control cells exposed to prolonged PTC or PTC:PD became sensitive to higher doses of these treatments (Figure 4D), likely linked to reduced CHD7 expression induced by the BMI inhibitor (Supplementary Figure S4A). Remarkably, the PTC:PD combination treatment overcame the single-agent acquired resistance in BMI1^High^;CHD7^Low^ cells (Figure 4E and Supplementary Figure S4B).

Our data show that BMI1 and MAPK/ERK combination treatment is anti-tumorigenic and does not induce acquired resistance, which is instead observed upon single agents’ treatment.

### BMI1 and MAPK/ERK Combination Treatment Improves Survival of BMI1^High^;CHD7^Low^ MB Xenografts

**Fig. 5:**
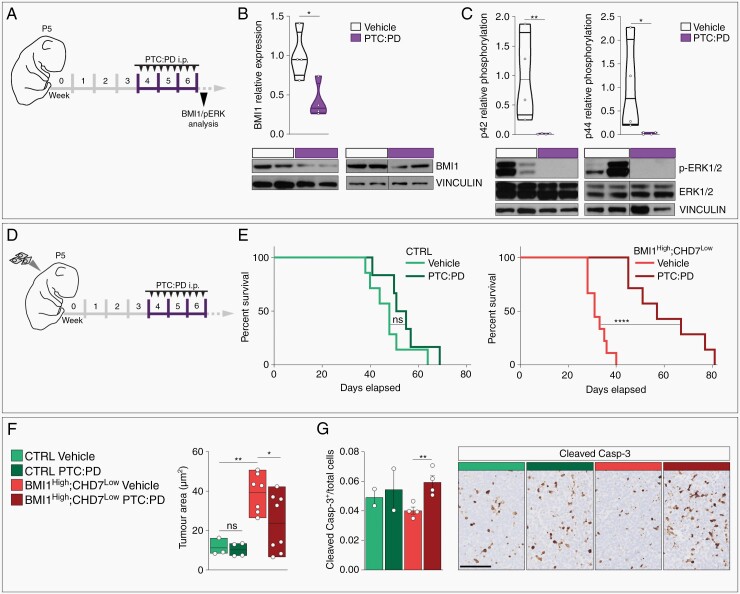
Anti-tumor effect of combined BMI1 and MAPK inhibition in MB is dependent on the BMI1^High^;CHD7^Low^ signature. A. Experimental design to assess the *in vivo* efficacy in the cerebellum of PTC:PD combined treatment, administrated through intra-peritoneal injection (i.p.). B–C. Western blot analysis and quantification of BMI1 (B) and phosphorylated/total ERK1/2 (p-ERK1/2) (C) in cerebellar protein extracts of mice treated with vehicle (white) or PTC:PD (violet). VINCULIN was used to normalize protein loading. *n* = 4 biological independent animal per group, paired *t*-test. D. Experimental design to assess the effect of PTC:PD on survival of mice xenografted with CHLA-01-Med MB cells. E. Kaplan-Meier survival curves of mice orthotopically xenografted with CTRL (green) or BMI1^High^;CHD7^Low^ (red) CHLA-01-Med MB cells treated with vehicle or PTC:PD. P-values determined by log-rank test. F. Quantification of tumor area in mice treated as described in D, one-way ANOVA. Number of biological independent animals per group is indicated. G. Quantification of fractions of cleaved caspase-3 positive cells of total number of tumor cells in mice treated as described in D. Number of biological independent animals per group is indicated, one-way ANOVA. All graphs report mean ± SEM. Scale bars = 100 µm.

Leveraging the evidence that PTC-209 inhibits GBM formation *in vivo*^28^ and that PD325901 blocks ERK phosphorylation in the ventral striatum^34^ when administrated intraperitoneally (i.p.), we set out to assess whether PTC:PD combination treatment could represent a novel therapeutic approach in a preclinical *in vivo* model of MB. First, we evaluated the effect of PTC:PD treatment, via i.p. injection, on their intended molecular targets in the naïve murine cerebellum with its intact blood-brain-barrier (BBB) (i.e. not xenografted with tumor forming cells) (Figure 5A). Analysis of cerebellar protein lysates showed a significant reduction of BMI1 expression and inhibition of ERK1/2 phosphorylation upon PTC:PD administration (Figure 5B,C), confirming that PTC and PD inhibit BMI1 and MAPK/ERK pathway *in vivo*. Next, control or BMI1^High^;CHD7^Low^ MB cells were injected in the cerebellum of new-born mice, which were treated with PTC:PD or vehicle administrated i.p. over three weeks (Figure 5D). Combined treatment significantly extended survival (Figure 5E and Supplementary Figure S5A,B,D) and decreased tumor size (Figure 5F and Supplementary Figure S5C) of mice xenografted with BMI1^High^;CHD7^Low^ MB cells, while no significant changes were observed in mice xenografted with control MB cells (Figure 5E,F and Supplementary Figure S5A,B,C,D). Mechanistically, PTC:PD treatment significantly increased apoptosis without impacting proliferation in BMI1^High^;CHD7^Low^ xenografts, as assessed by cleaved caspase-3 (Figure 5G) and ki-67 immunostaining (Supplementary Figure S5E) respectively, in keeping with cytotoxic rather than cytostatic effect.

In conclusion, our data confirm PTC:PD anti-tumor effects *in vivo* and provide preclinical evidence that BMI1 and MAPK/ERK could be targeted in patients with BMI1^High^;CHD7^Low^ MB.

### BMI1^High^;CHD7^Low^ Molecular Signature Characterizes G4 MB Patients With Enhanced MAPK/ERK Phosphorylation Activity

**Fig. 6:**
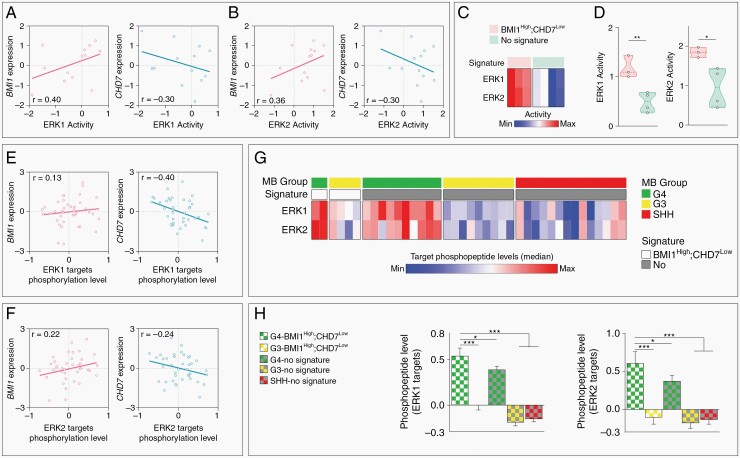
BMI1^High^;CHD7^Low^ signature predicts activation of MAPK/ERK phosphorylation pathway in G4 MB patients. A–B. Dot plots of correlations between *BMI1* or *CHD7* expression level and ERK1 or ERK2 kinase activity scores in MB patients. C–D. Heatmap (C) and violin plots (D) representing ERK1 and ERK2 kinase activity scores in MB patients with (light red, *n* = 3) or without (light green, *n* = 4) BMI1^High^;CHD7^Low^ signature, unpaired t-test. E–F. Dot plots of correlations between *BMI1* or *CHD7* expression level and ERK1 or ERK2 target phosphopetides levels in MB patients. G. Heatmap representing median value of all phosphopetides targeted by ERK1 (*n* = 73) or ERK2 (*n* = 21) in BMI1^High^;CHD7^Low^ G4 (*n* = 2) or G3 (*n* = 4) MB patients and G4 (*n* = 10), G3 (*n* = 9) or SHH (*n* = 14) MB patients without the signature. H. Histograms of the level of all phosphopeptide targeted by ERK1 or ERK2 in MB patients with or without BMI1^High^;CHD7^Low^ signature as shown in G, one-way ANOVA. All graphs report mean ± SEM.

To explore the value of BMI1^High^;CHD7^Low^ molecular signature as a potential predictive marker of response in future clinical trials, we analyzed a cohort of MB patients where proteogenomic analysis had been performed on surgically resected tumor samples.^35^ Analysis of ERK1 and ERK2 kinase activity scores obtained from phospho-proteomic data showed a positive and negative correlation with *BMI1* and *CHD7* expression level respectively, suggesting a role for the CHD7-BMI1-MAPK regulatory axis in patients’ tumor samples as well (Figure 6A,B). Next, we identified BMI1^High^;CHD7^Low^ MB and found an increased ERK1 and ERK2 activity compared to samples without the signature (Figure 6C,D). Interestingly, BMI1^High^;CHD7^Low^ MB samples show expression of gene markers consistent with G3 and G4 MB (Supplementary Figure S6A,B), indicating a possible subgroup-specific link between BMI1/CHD7 and MAPK activity. Notably, phospho-proteomic studies of MB samples identified activation of MAPK/ERK signaling in G4.^21,36^ Hence, we analyzed BMI1/CHD7 expression and ERK1 and ERK2 phosphopeptides level in a second independent proteogenomic study performed on a cohort of primary tumors classified into MB subgroups.^36^ First, we confirmed a positive correlation between *BMI1* expression and ERK1/2 phosphorylation while the latter correlated negatively with *CHD7* levels (Figure 6E,F). Next, we found increased ERK1 and ERK2 phosphopeptide levels in BMI1^High^;CHD7^Low^ G4 when compared to samples without the signature of all available subgroups (Figure 6G,H). Strikingly, a significant increase of phosphopeptide level of ERK1 and ERK2 was found also comparing BMI1^High^;CHD7^Low^ G4 with G3 with the same signature (Figure 6H), suggesting a relevance of the BMI1^High^;CHD7^Low^ specifically in G4 MB subgroup. Finally, we analyzed transcriptomic data of MB samples,^1^ divided in subgroups and stratified according to BMI1^High^;CHD7^Low^ molecular signature (Supplementary Figure S6C), and obtained ssGSEA scores of a MEK activation signature comprising 18 genes (Supplementary Figure S6D), the expression of which provides an estimate of the MAPK/ERK functional output in lung, colon and melanoma tumors.^37^ We found a significant negative score when analyzing all MB or G4 MB with a BMI1^High^;CHD7^Low^ signature compared to samples without the signature (Supplementary Figure S6E). These results are in line with previous reports describing a discordance between RNA and protein levels particularly in G4 MB, and indicate that MAPK pathway activation can be inferred exclusively from phospho-proteomic data.^21,36^

In conclusion, our data suggest that BMI1^High^;CHD7^Low^ molecular signature is a predictive marker of MAPK/ERK activation in G4 MB patients.

## Discussion

Here we identify a synergistic vulnerability of BMI1^High^;CHD7^Low^ MB cells to combined treatment with BMI1 and MAPK/ERK inhibitors. We show that BMI1 binds MAPK-regulated genes specifically when CHD7 is expressed and that the CHD7-BMI1-MAPK regulatory axis underpins the anti-tumor activity of BMI1 and MAPK/ERK inhibitors *in vitro* and in an *in vivo* preclinical model of BMI1^High^;CHD7^Low^ MB where it significantly extends survival of xenografted mice. Finally, we observe that BMI1^High^;CHD7^Low^ signature characterizes G4 MB patients with an enhanced ERK1 and ERK2 phosphorylation activity.

BMI1 plays a key role not only in maintaining tumor growth but also in recurrence and therapy resistance in a wide variety of tumors, including MB.^9,11,15,32^ Hence, the therapeutic potential of BMI1 targeting has been widely investigated with many studies describing anti-tumor activity of the inhibitor PTC-209 in different tumors such as DIPG^14^ and rhabdomyosarcoma.^38^ We show that BMI1^High^;CHD7^Low^ MB cells are more responsive to PTC treatment while non-neoplastic NSC are not affected by the treatment, as previously reported for other normal/healthy cells,^29^ raising the possibility that the treatment would have negligible impact on healthy progenitors in the developing cerebellum.

Epigenetic modifications are reversible changes, rendering therapeutic inhibition of their regulatory factors attractive; for example, the BMI1 inhibitor PTC-596 is currently in Phase 1 clinical trials^39^ in adults with solid tumors, such as leiomyosarcoma and in children with DIPG and high grade glioma. However, acquired resistance to drugs inhibiting epigenetic modifiers is emerging^33^ and combination therapies that account for evolutionary genomic, epigenomic, and transcriptomic modifications not directly linked to the acute response induced by the epigenetic drug must be explored. Analysis of PTC-treated BMI1^High^;CHD7^Low^ MB cells, revealed an additional regulatory layer of the MAPK/ERK signaling pathway. We had previously reported that the molecular convergence of BMI1 and CHD7 on ERK pathway led to its overactivation.^9^ Here, we identified MAPK-related genes as BMI1-direct targets dependent on CHD7 expression. In particular, we observed an enrichment in motifs recognized by ETS transcription factors, known MAPK substrates,^17^ on promoters bound by BMI1 specifically when CHD7 is expressed. CHD7 mobilizes and relocates nucleosomes and it can function both as an activator or repressor.^40^ Consistently, we previously described an increased accessibility of the DUSP4 locus upon CHD7 silencing.^9^ Here, we describe an additional and complementary regulation by which CHD7 contributes to BMI1 binding to MAPK-dependent chromatin loci, further elucidating the molecular mechanisms underpinning the BMI1 and CHD7 convergence.

In keeping with the previously observed overactivation of ERK phosphorylation upon CHD7 silencing,^9^ we demonstrate that BMI1^High^;CHD7^Low^ MB cells rely on MAPK/ERK pathway for their cell growth and survival and are therefore more responsive to its inhibition. A wide range of MAPK/ERK inhibitors are currently used in cancer therapies^41^ including PD325901, used in the present study, which is in Phase 1 and 2 clinical investigations. Furthermore, inhibitors of BRAF/MEK are used in clinical trials for pediatric brain tumors^42^; hence, a repurposing of MAPK/ERK in MB treatment could be envisaged.

Cancer treatment frequently benefits from combination therapy, which targets multiple pathways to obtain a superior effect while reducing the development of drug-resistance. Acquired resistance to MAPK inhibitors has been described in multiple cancers and can emerge from the reactivation of MAPK signaling, activation of parallel pathways sustaining tumor growth, but also from tumor transformation mediated by changes in phenotypes or rewiring of metabolism. Beside genomic and transcriptomic alterations, multiple epigenetic mechanisms are associated to resistance to MAPK inhibitors treatment.^43,44^ Therefore, therapeutic approaches with compounds targeting epigenetic factors in synergy with MAPK inhibitors are gaining interest, with studies suggesting a superior anti-cancer effect and, importantly, potential to overcome therapy resistance upon synergistic treatment.^45,46^ Here, we demonstrate the efficacy of targeting BMI1 and MAPK/ERK individually in BMI1^High^;CHD7^Low^ MB cells, and importantly we show that the two inhibitors synergize in these cells and their combination circumvents acquired resistance to both single and combined treatments. We demonstrate in drug interaction studies that BMI1 and MAPK/ERK inhibitors act in a strong synergy even when used at lower concentrations, suggesting that the combination therapy could be well tolerated. Interestingly, we show that prolonged inhibition of BMI1 or BMI1 and MAPK/ERK reduces CHD7 expression and sensitizes MB cells to the combination therapy, raising the possibility that a larger number of patients could be amenable to this therapeutic approach.

We show that PTC-209 and PD325901 extend survival of BMI1^High^;CHD7^Low^ MB xenografts by increasing apoptosis. Induction of apoptosis by PTC-209 or PD325901 administration has been previously reported in other tumors^29,38,47,48^ both *in vitro* and *in vivo*. Interestingly, a recent study suggested that the new generation BMI1 inhibitor PTC-596 could be more effective in mediating apoptosis in AML when combined with the MEK inhibitor trametinib.^49^ Both PTC-209 and PTC-596 induce a decrease in BMI1 protein level, by interfering with post-translational mechanisms or via phosphorylation events that induce accelerated degradation^29,50^ respectively. Further investigation will be needed to explore the value of this new generation of BMI1 inhibitors in MB.

Independent integrated proteogenomic analysis in MB revealed lack of concordance between differential RNA expression and the corresponding changes at the (phospho)protein level.^21,36^ Thus, (phospho)proteomic data can reveal mechanisms that would have not been identified with transcriptome analysis. Intriguingly, increased activation of MAPK/ERK signaling in G4 MB can exclusively be inferred by (phospho)proteomic studies, while RNA-Seq data predicts an opposite effect.^21^ However, transcriptomic data are more widely available than phospho-proteomic, and they are now used, together with DNA methylation, to refine the diagnosis and molecularly stratify MB patients. Thus, the identification of biomarkers based on RNA expression could be readily implemented in clinical diagnostic. Interestingly, we found that stratification of MB patients on the basis of a BMI1^High^;CHD7^Low^ molecular signature (obtained from RNA-Seq) identifies increased ERK1/2 activity at phospho-proteomic level in the G4 subgroup.

In conclusion, molecular dissection of the CHD7-BMI1-MAPK regulatory axis in BMI1^High^;CHD7^Low^ MB identifies this signature as a proxy to predict MAPK functional activation, which can be effectively drugged in preclinical models, and paves the way for further exploration of combined BMI1 and MAPK targeting in G4 MB patients.

## Supplementary Material

noac052_suppl_Supplementary_Material
