# Supplementary material for: Combination of BMI1 and MAPK/ERK inhibitors is effective in medulloblastoma
Source: Neuro Oncol. 2022 Feb 25;24(8):1273–85. doi: 10.1093/neuonc/noac052 (PMC9340634; doi:10.1093/neuonc/noac052)
Supplement: noac052_suppl_Supplementary_Material [file noac052_suppl_supplementary_material.pdf]

Supplementary Materials for

Combination of BMI1 and MAPK/ERK inhibitors is effective in medulloblastoma.

Sara Badodi*, Nicola Pomella, Yau Mun Lim, Sebastian Brandner, Gillian Morrison, Steven M. Pollard, Xinyu Zhang, Nicolae Radu Zabet, Silvia Marino*

*Corresponding authors. Emails: s.badodi@qmul.ac.uk, s.marino@qmul.ac.uk

This PDF file includes:

Supplementary Methods

Code availability

Data availability

Supplementary References

Figs. S1 to S6

Supplementary Methods

Cell culture conditions

ICb1299 patient-derived MB lines were obtained from Dr Xiao-Nan Li, Baylor College of Medicine, Texas Children Cancer Centre, USA[1]. ICb1299 were cultured in DMEM (high glucose, GlutaMAX™, ThermoFisher) supplemented with 10% foetal bovine serum (FBS, Gibco) and 1% Penicillin-Streptomycin (Gibco). CHLA-01-Med MB cells were purchased from ATCC (CRL3021) and cultured as described previously[2]. Briefly, cells were grown in DMEM-F12 (Gibco) supplemented with B27 (Gibco), 20 ng/mL human epidermal growth factor (EGF, Peprotech) and 10 ng/mL human basic fibroblast growth factor (FGF, Peprotech). MB subtype identity for the two cell lines has been previously shown[1;3]. Human foetal NSC lines were obtained from the Cancer Research UK-funded Glioma Cellular Genetics Resource (www.gcgr.org.uk) and cultured as previously described[4]. Cells were maintained at 37ºC and were sub-cultured every 3 days once they reached confluence.

Production of shRNA lentiviral vector, gene silencing and CHD7 overespression

GIPZ lentiviral shRNA vectors containing a hairpin sequence targeting CHD7 and the coding sequences for GFP and puromycin resistance gene were purchased from Dharmacon, UK. Packaging, virus production and determination of titre were carried out as previously reported[3]. Cells were infected overnight at a multiplicity of infection (MOI) of 1. After 96h from the infection, puromycin selection at a concentration of 2.5 µg/ml was applied to enrich for the transduced population. The efficacy of gene silencing was assessed by RT-qPCR or Western blot analysis. CHD7 overespression was obtained as previously described[3]. Cells were transfected with pcDNA-FLAG-His-CHD7-WT (kindly provided by Albert Basson) with Lipofectamine 3000 (Invitrogen) following manufacturer's instructions. Briefly, $1x10^6$ MB cells were reversed transfected in 1mL of Opti-MEM medium (Gibco) with 4µg of DNA using 5.5 µL of Lipofectamine 3000 Reagent and 8 µL of P3000 Reagents. Given the low efficiency of transfection in MB cells, the large size of CHD7 expressing vector and the high molecular weight of the encoded protein, overespression of CHD7 was analysed by RT-qPCR 48h after transfection.

RNA extraction and RT-qPCR analysis

Total RNA was isolated from cell pellets with RNeasy Micro purification kit (Qiagen) and digested with DNaseI (Applied Biosystems). The cDNA synthesis was carried out with SuperScript III Reverse Transcriptase Kit (Invitrogen) following manufacturer's protocol. Analysis of gene expression was performed with the Applied Biosystems 7500 Real-Time PCR System using SYBR Green PCR Master Mix (Applied Biosystems) according to standard protocols. Technical triplicates for each sample were analyzed. The Ct values of all the genes analyzed were normalized to the average Ct of *ACTB* and *ATP5F1B* and fold changes were calculated. Primers used in SYBR Green qPCR are the following.

ACTB: FW 5'-GCGAGAAGATGACCCAGATC-3', REV 5'-CCAGTGGTACGGCCAGAGG-3'

ATP5F1B: FW 5'-CCCAGGCTGGTTCAGAGGT-3', REV 5'-AGGGGCAGGGTCAGTCAAG-3'

CHD7: FW 5'-GAAGAAGATATAGAGACCCCAC-3', REV 5'-TCTTTGGTACATAACTTGGC-3'

Cell viability, growth and drug interaction assays

For growth curve and cell viability assays MB cells or hNSCs were seeded in 24-well plates at the same density. Cells were treated with PTC-209 (Tocris) and PD325901 (Tocris) both dissolved in DMSO (Sigma) at the indicated concentrations. At specific time points and after appropriate treatment, cells were harvested, and the number of viable cells was counted with a hemocytometer and Trypan Blue staining or with CyQUANT Direct Red Cell Proliferation Assay Kit (ThermoFisher Scientific). Synergy/antagonist effect of the combined treatment was identified by Loewe Model with Combenefit software[5]. Loewe model describes the expected effect of a combination of two drugs as if one single drug was combined with itself. Hence, synergy is achieved if the effect obtained with the combination of the two drugs is greater than the effect of either drug alone[6]. Combination index values were determined with CompuSync tool and calculated with Chou-Talalay method[7].

Western blot analysis

MB cells or hNSCs were lysed for 30 minutes on ice using RIPA lysis buffer supplemented with 2mM PMSF, 1mM sodium orthovanadate and protease inhibitor cocktail (PIC) (RIPA Lysis Buffer System,

Santa Cruz) followed by 3 pulses of sonication to obtain total protein lysate. Nuclear and cytoplasmic fractions of cells were obtained with two different lysis buffers as described previously[8]. Briefly, cells were harvested in Buffer A (10 mM HEPES pH 7.9, 10 mM KCl, 0.1 mM EDTA, 0.15% Nonidet P40 (NP40) and 0.1 mM EGTA) supplemented with 1 mM DTT and PIC. Cells were homogenized through a 26G needle, nuclei were isolated by centrifugation and the supernatant (cytoplasmic fraction) was collected. Nuclei were washed quickly with ice-cold PBS, suspended in Buffer B (20 mM HEPES pH 7.9, 400 mM NaCl, 1 mM EDTA, 1 mM EGTA and 0,5% NP 40) supplemented with 1 mM DTT and PIC and lysed by sonication. Protein concentration was determined using BCA Protein Assay Kit (Pierce). Equal amounts of protein were separated by SDS-PAGE and transferred onto nitrocellulose membrane (Amersham). After transfer, the membrane was blocked for one hour at room temperature in 5% skimmed milk in TBST buffer (25 mM TrisHCl, 137 mM NaCl, 0.1% Tween 20, pH 7.5) and probed with different antibodies. Incubation with primary antibody was performed overnight at 4°C followed by appropriate secondary HRP- conjugated antibodies (anti-rabbit IgG or anti-mouse IgG, 1:5000, Amersham) for one hour at room temperature. Enhanced chemiluminescence (ECL Plus, Amersham) was used for detection of the bands. The following primary antibodies were used: mouse monoclonal anti-BMI1 (1:1000, clone AF27, Active Motif), anti-ubiquityl-Histone H2A (1:200, E6C5, Millipore) and anti-Vinculin (1:5000, V4505, Sigma); rabbit monoclonal anti-p44/42 MAPK (Erk1/2) (1:1000, 137F5, Cell Signaling), anti-phospho-p44/42 MAPK (Erk1/2) (Thr202/Tyr204, 1:1000, D13.14.4E, Cell Signaling); rabbit polyclonal anti-CHD7 (1:500, ab117522, abcam), anti-Histone H3 (1:500, 07-690, Millipore) and goat polyclonal anti-Lamin B (1:5000, sc-6216, Santa Cruz).

RNA sequencing (RNA-Seq) analysis

Total RNA was extracted with RNA/DNA/Protein Purification Plus kit (Norgen), polyA mRNA was selected and library prepared using the NEBNext Ultra II Directional RNA Library Prep kit. Libraries were size selected, multiplexed and 75bp paired-end sequences were obtained with HiSeq4000 (Illumina). Quality control was performed with FastQC and adapter sequences were removed using Trimgalore v0.6.5 (www.bioinformatics.babraham.ac.uk/projects/). Reads were then aligned to the Ensembl GRCh38 human reference genome using STAR v2.6.1[9], with gene count quantification mode, retaining only uniquely mapped reads. Lowly expressed genes (CPM < 1) were filtered out

via a custom-made script in R and further biotype filtering was performed in R with the Bioconductor packages NOISeq[10] and biomaRt[11], removing residual highly expressed mitochondrial and ribosomal RNA. Trimmed mean of M-values (TMM) normalization was applied to the dataset prior to differential expression (DE) analysis, performed using the Bioconductor package edgeR[12]. Specifically, a negative binomial generalized log-linear model (glmfit) was fitted to the read counts and the likelihood ratio test (glmLRT) was conducted for each comparison of interest. The Benjamini-Hochberg FDR cut-off was set at 0.05. The versions of all relevant Bioconductor packages were compatible with R v3.5.3.

Pathway and gene set analysis

Ingenuity Pathway Analysis (IPA, Qiagen), Reactome[13] and Panther Gene Ontology[14] tools were used to assess biological pathways and gene ontology terms that showed significant enrichment in the various gene sets. Functional analysis of significant 24h PTC-mediated DEG lists was carried out with IPA software with Canonical Pathways tool following the manufacturer standard protocol. Reactome Pathway Database was used to interrogate dataset of 48h-mediated DEG lists, while Panther Gene Ontology was used to analyse GO terms significantly enriched in RNA-Seq analysis. The enrichment for each term was deemed statistically significant if the adjusted p-value (FDR) was lower than 0.05. Cytoscape v.3.7.2[15] was used to visualise relevant biological networks of enriched pathways, together with EnrichmentMap and AutoAnnotate applications. Several layout parameters were tuned to achieve the current Cytoscape visualization.

ssGSEA (single sample Gene Set Enrichment Analysis)[16] was used to calculate MEK activation scores from MB transcriptomic data. The function gsva in the GSEA package from the R Bioconductor repository was used to compute ssGSEA scores, with “method=ssgsea” and default settings.

ChIP-Seq analysis

The ChIP-Seq data used in this study were selected from GSE156077[17]. Briefly, reads were first pre-processed with TrimGalore and aligned to the human reference genome GRCh38 via Bowtie v.2.3.4 [18], then peaks were called with MACS2[19] with a minimum fold enrichment of 2 and FDR<0.05. The

R Bioconductor package ChIPseeker[20] was chosen to perform the annotation of the ChIP-Seq peaks. Specifically, the function annotatePeak determined the overlap between the peak and transcription start site (TSS) of the nearest gene, assigning a category to each peak, with the following priority if more than one categories overlapped: promoter, 5' UTR, 3' UTR, exon, intron and distal intergenic. The latter was merged with the so-called downstream regions. The annotation was based on the current version of the human transcriptome (hg38). The TSS region range was set from -3000 to 3000 base pairs, which is the default setting[20].

Motif Enrichment Analysis and Peak Annotation

The Motif Enrichment analysis was performed with Homer[21]. For each comparison, a "foreground" list of regions (BMI1-bound peaks on promoters of gene regulated by PTC-209 in CTRL MB cells) was compared to a "background" list (BMI1-bound peaks on promoters of gene regulated by PTC-209 in BMI1^{High};CHD7^{Low} MB cells), to determine motifs enriched in the foreground. Specifically, the tool findMotifsGenome.pl looked for significantly enriched motifs (p-value < 0.05) with a length spanning a wide range of standard values (6,8,10,12,15,20,25,30,35,40,45,50 bp) in a region of default size (200 bp) at the centre of each sequence. The tool performed a *de novo* search as well as checked the enrichment of known motifs using a binomial distribution. To minimize the bias towards long repeats in the genome, we used the option -mask. In addition, to improve the sensitivity of the algorithm, the maximum number of mismatches allowed in the global optimization phase was set to 3. In the context of *de novo* search, a scoring algorithm assigned a ranked list of best matches (known motifs or genes) to each de novo motif, to inform the biological interpretation of the results.

Phospho-proteomic data analysis

Phospho-proteomic data were retrieved from previously published studies and ERK1 and ERK2 kinase scores[22] or levels of phosphopeptides matched to ERK1 and ERK2[23] were used. Expression gene markers analysis for specific MB subgroup was performed using gene lists previously described[24;25] and clustering of samples in corresponding heatmaps was obtained with Morpheus web tool (https://software.broadinstitute.org/morpheus). Classification of patients based on BMI1^{High};CHD7^{Low} signature was performed as previously described[3].

Identification of tumour area and Ki67 or cleaved Casp-3 positive cells

Image analysis was performed using QuPath[26]. Machine learning-based pixel classifiers were trained to identify tumour regions in the sections. For Ki67, regions with high DAB staining at a very low resolution (14.75 µm/px) were used to train the classifier to detect tumour. Tumour regions with no Ki67 staining that were missed by the pixel classifier were manually filled in. For Casp-3, regions with higher haematoxylin staining intensity (i.e. regions with high cellularity) at a low resolution (7.05 µm/px) were used to train the classifier to detect tumour. As this also detects the cerebellar granular layer, these regions were manually excluded. For Ki67 and Casp-3, non-specific regions of interest (ROI) due to artefacts (e.g. tissue folding, dust shadows) were removed.

To identify Ki67 and Casp-3 positive cells, all individual nuclei (and hence cells) in the tumour regions were first detected by haematoxylin and a watershed-based method. Machine learning-based object classifiers were then trained and applied to distinguish DAB-positive cells for Ki67 and Casp-3 from DAB-negative cells.

Code availability

The custom-made code used in this study is available in GitHub repository (https://github.com/nickpom88/rnaseq/blob/main/filter_genes_np2.R).

Data availability

The authors declare that all the data supporting the findings of this study are available within the Article and its Supplementary Information files. The datasets are available in the NCBI Gene Expression Omnibus database (GSE172363) or are available from the corresponding author upon reasonable request.

Publicly available datasets used in the study: GSE156077 (αBMI1-ChIPSeq data), GSE85217 (Expression data from primary medulloblastoma samples), Proteogenomic data for brain tumour samples (https://cptac-data-portal.georgetown.edu/study-summary/S047), Reactome (https://reactome.org/), Panther Gene Ontology tool (http://geneontology.org/).

Supplementary References

1. Shu, Q *et al.* Direct orthotopic transplantation of fresh surgical specimen preserves CD133+ tumor cells in clinically relevant mouse models of medulloblastoma and glioma. *Stem Cells* **26**, 1414-1424 (2008).
2. Badodi, S *et al.* Establishment and Culture of Patient-Derived Primary Medulloblastoma Cell Lines. *Methods Mol Biol* **1869**, 23-36 (2019).
3. Badodi, S *et al.* Convergence of BMI1 and CHD7 on ERK Signaling in Medulloblastoma. *Cell Rep* **21**, 2772-2784 (2017).
4. Conti, L *et al.* Niche-independent symmetrical self-renewal of a mammalian tissue stem cell. *PLoS Biol* **3**, e283 (2005).
5. Di Veroli, GY *et al.* Combenefit: an interactive platform for the analysis and visualization of drug combinations. *Bioinformatics* **32**, 2866-2868 (2016).
6. Lederer, S *et al.* Additive Dose Response Models: Defining Synergy. *Front Pharmacol* **10**, 1384 (2019).
7. Chou, TC *et al.* Quantitative analysis of dose-effect relationships: the combined effects of multiple drugs or enzyme inhibitors. *Adv Enzyme Regul* **22**, 27-55 (1984).
8. Badodi, S *et al.* Phosphorylation-dependent degradation of MEF2C contributes to regulate G2/M transition. *Cell Cycle* **14**, 1517-1528 (2015).
9. Dobin, A *et al.* STAR: ultrafast universal RNA-seq aligner. *Bioinformatics* **29**, 15-21 (2013).
10. Tarazona, S *et al.* Data quality aware analysis of differential expression in RNA-seq with NOISeq R/Bioc package. *Nucleic Acids Res* **43**, e140 (2015).
11. Durinck, S *et al.* Mapping identifiers for the integration of genomic datasets with the R/Bioconductor package biomaRt. *Nat Protoc* **4**, 1184-1191 (2009).
12. Robinson, MD *et al.* edgeR: a Bioconductor package for differential expression analysis of digital gene expression data. *Bioinformatics* **26**, 139-140 (2010).
13. Wu, G *et al.* Functional Interaction Network Construction and Analysis for Disease Discovery. *Methods Mol Biol* **1558**, 235-253 (2017).
14. Mi, H *et al.* PANTHER in 2013: modeling the evolution of gene function, and other gene attributes, in the context of phylogenetic trees. *Nucleic Acids Res* **41**, D377-386 (2013).
15. Shannon, P *et al.* Cytoscape: a software environment for integrated models of biomolecular interaction networks. *Genome Res* **13**, 2498-2504 (2003).
16. Barbie, DA *et al.* Systematic RNA interference reveals that oncogenic KRAS-driven cancers require TBK1. *Nature* **462**, 108-112 (2009).
17. Badodi, S *et al.* Inositol treatment inhibits medulloblastoma through suppression of epigenetic-driven metabolic adaptation. *Nat Commun* **12**, 2148 (2021).
18. Langmead, B *et al.* Fast gapped-read alignment with Bowtie 2. *Nat Methods* **9**, 357-359 (2012).

19. Zhang, Y *et al.* Model-based analysis of ChIP-Seq (MACS). *Genome Biol* **9**, R137 (2008).
20. Yu, G *et al.* ChIPseeker: an R/Bioconductor package for ChIP peak annotation, comparison and visualization. *Bioinformatics* **31**, 2382-2383 (2015).
21. Heinz, S *et al.* Simple combinations of lineage-determining transcription factors prime cis-regulatory elements required for macrophage and B cell identities. *Mol Cell* **38**, 576-589 (2010).
22. Petralia, F *et al.* Integrated Proteogenomic Characterization across Major Histological Types of Pediatric Brain Cancer. *Cell* **183**, 1962-1985 e1931 (2020).
23. Archer, TC *et al.* Proteomics, Post-translational Modifications, and Integrative Analyses Reveal Molecular Heterogeneity within Medulloblastoma Subgroups. *Cancer Cell* **34**, 396-410 e398 (2018).
24. Cho, YJ *et al.* Integrative genomic analysis of medulloblastoma identifies a molecular subgroup that drives poor clinical outcome. *J Clin Oncol* **29**, 1424-1430 (2011).
25. Northcott, PA *et al.* Subgroup-specific structural variation across 1,000 medulloblastoma genomes. *Nature* **488**, 49-56 (2012).
26. Bankhead, P *et al.* QuPath: Open source software for digital pathology image analysis. *Sci Rep* **7**, 16878 (2017).

Figure S1

Badodi et al

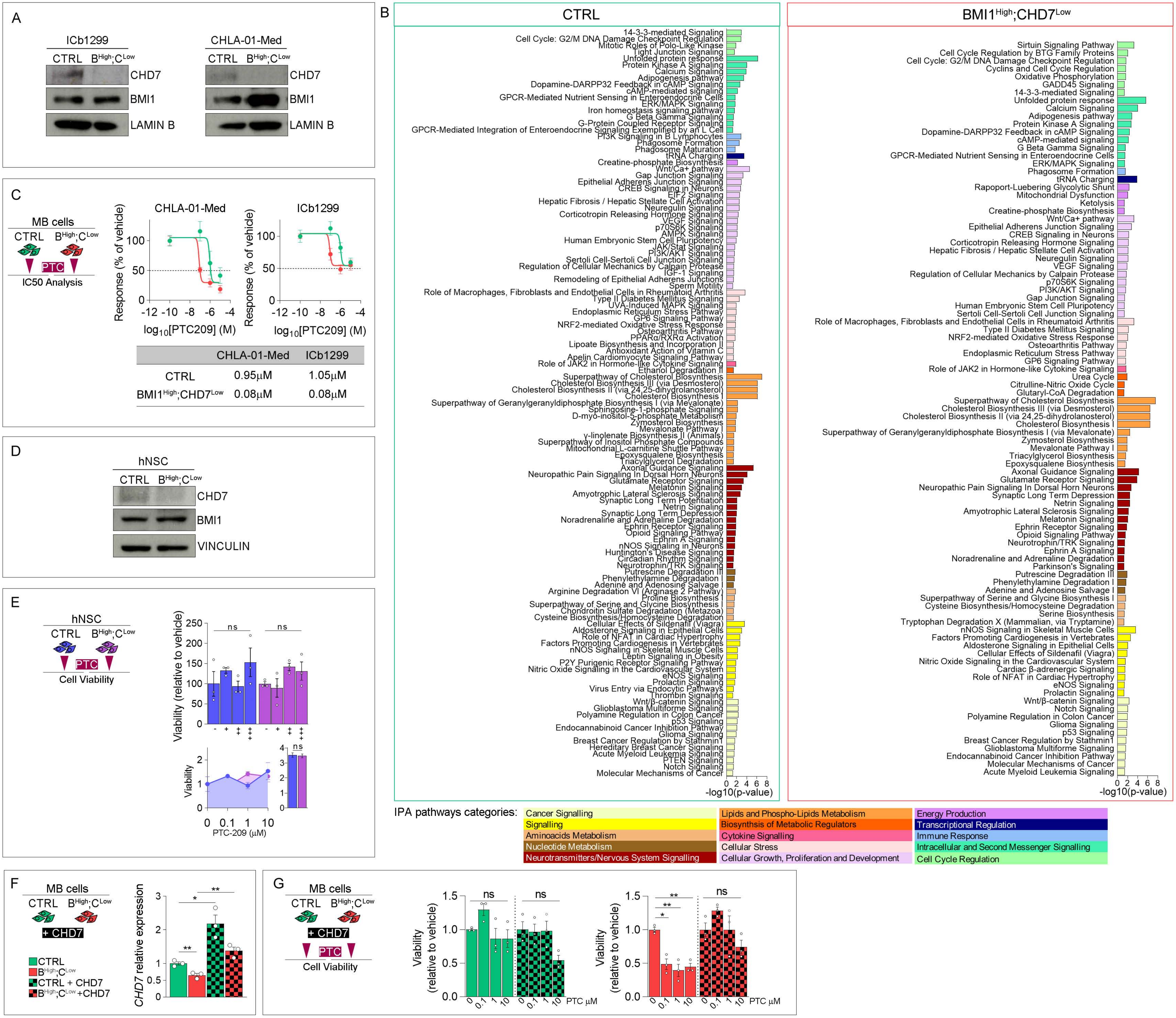

Fig. S1. CHD7 sensitizes MB cells, but not hNSC, to BMI1 inhibition.

A. Representative images of Western blot analysis of BMI1 and CHD7 expression in nuclear extracts of ICb1299 and CHLA-01-Med MB cells. LAMIN B was used to normalize nuclear protein loading.

B. Histograms showing -log10(FDR) values of canonical pathways differentially enriched between PTC- or vehicle-treated control (CTRL) and $BMI1^{High}$;$CHD7^{Low}$ MB cells in RNA-Seq analysis. Canonical pathways are classified based on IPA categories list and colour coded accordingly.

C. Dose-response curves of CTRL (green) or $BMI1^{High}$;$CHD7^{Low}$ (B^{High};C^{Low}, red) CHLA-01-Med and ICb1299 MB cells upon treatment with increasing concentrations of PTC-209. IC50 doses for each cell lines and conditions are reported in the table. n=5 independent biological experiments for CHLA-01-Med or n=8 for ICb1299.

D. Representative images of Western blot analysis of BMI1 and CHD7 expression in control (CTRL) and $BMI1^{High}$;$CHD7^{Low}$ ($BMI1^{H}$;$CHD7^{L}$) hNSC. VINCULIN was used to normalize protein loading.

E. Cell viability assays of CTRL (violet) or $BMI1^{High}$;$CHD7^{Low}$ (purple) hNSC cells upon treatment with increasing concentrations of PTC-209. Histograms represent percentages of viable cells relative to vehicle-treated (DMSO) cells (top). Measurement of Area-under-Curve (AUC) and histograms representing mean AUC were used to evaluate the overall response to treatment (bottom). n=3 independent biological experiments, two-way ANOVA.

F. RT-qPCR analysis showing quantification of *CHD7* expression in CTRL and $BMI1^{High}$;$CHD7^{Low}$ CHLA-01-Med MB cells upon CHD7 overexpression. n=3 biological independent experiments, one-way ANOVA.

G. Cell viability assays of CTRL or $BMI1^{High}$;$CHD7^{Low}$ CHLA-01-Med MB cells upon CHD7 overexpression and treatment with increasing concentrations of PTC-209. Histograms represent percentages of viable cells relative to vehicle-treated (DMSO) cells. n=3 independent biological experiments, two-way ANOVA.

All graphs report mean ± SEM.

Figure S2

Badodi et al

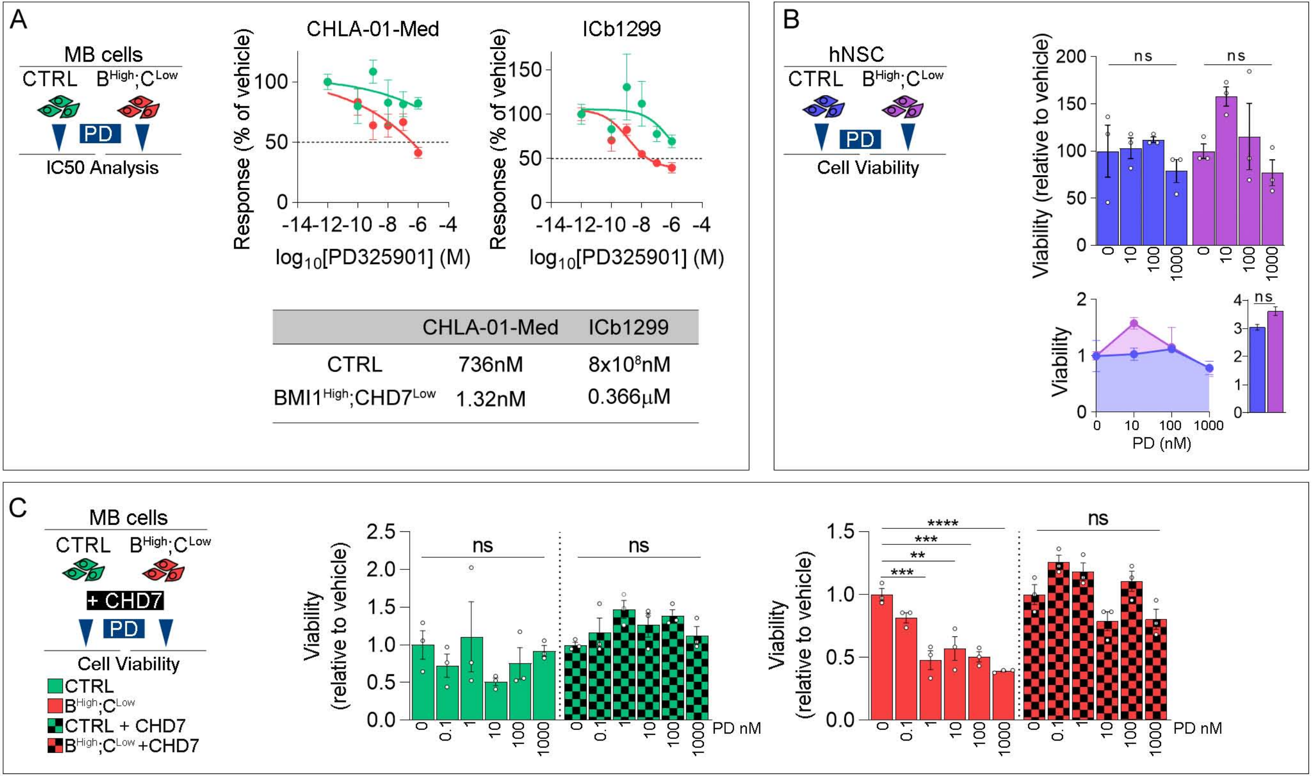

	CHLA-01-Med	ICb1299
CTRL	736nM	$8x10^8$nM
BMI1^{High};CHD7^{Low}	1.32nM	0.366μM

Fig. S2. BMI1^{High};CHD7^{Low} MB cells, but not hNSC, are more responsive to MAPK inhibition.

A. Dose-response curves of control (CTRL, green) or BMI1^{High};CHD7^{Low} (B^{High};C^{Low}, red) CHLA-01-Med and ICb1299 MB cells upon treatment with increasing concentrations of PD329501. IC50 doses for each cell lines and conditions are reported in the table. n=7 independent biological experiments.

B. Cell viability assays of CTRL (violet) or BMI1^{High};CHD7^{Low} (purple) hNSC cells upon treatment with increasing concentrations of PD. Histograms represent percentages of viable cells relative to vehicle-treated (DMSO) cells (top). Measurement of Area-under-Curve (AUC) and histograms representing mean AUC were used to evaluate the overall response to treatment (bottom). n=3 independent biological experiments, two-way ANOVA.

C. Cell viability assays of CTRL or BMI1^{High};CHD7^{Low} CHLA-01-Med MB cells upon CHD7 overexpression and treatment with increasing concentrations of PD325901. Histograms represent percentages of viable cells relative to vehicle-treated (DMSO) cells. n=3 independent biological experiments, two-way ANOVA.

All graphs report mean ± SEM.

Figure S3 Badodi et al

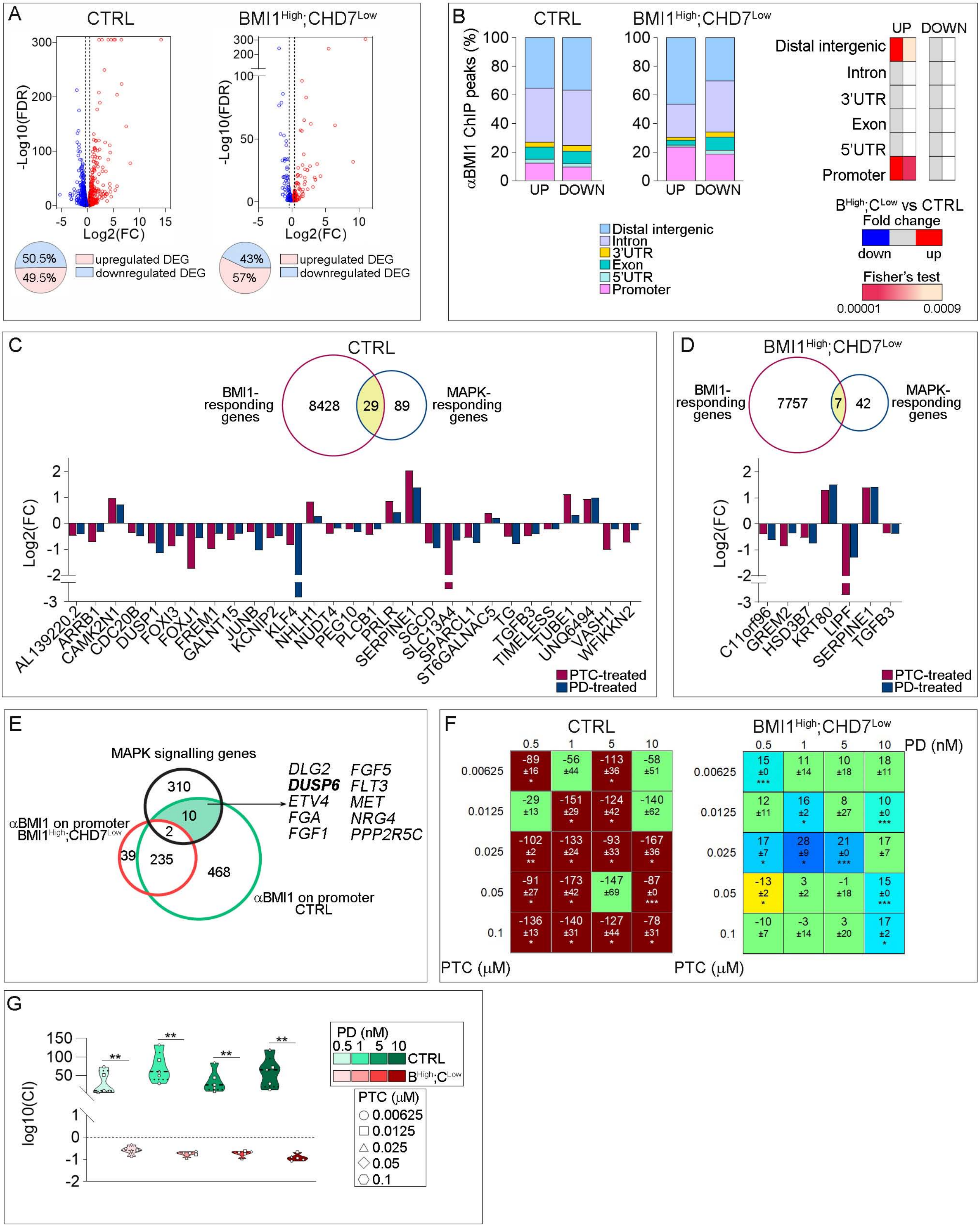

Fig. S3. CHD7-BMI1-MAPK regulatory axis in MB cells.

A. Volcano plots of BMI1 direct targets in control (CTRL) or BMI1^{High};CHD7^{Low} CHLA-01-Med and ICb1299 MB cells (identified in Figure 3A) and direction of regulation upon PTC-treatment (top). Red and blue dots represent genes with Log2FC >0 or <0 and FDR<0.05 respectively. Pie charts representing percentages of PTC-upregulated or downregulated BMI1 direct targets in CTRL or BMI1^{High};CHD7^{Low} (bottom).

B. Distribution of genomic annotations of ChIP-Seq peaks of BMI1 targets identified in A (left). Heatmaps represent Fisher's exact test statistical analysis of fold changes in percentages of BMI1 ChIP-peaks upon BMI1^{High};CHD7^{Low} modelling (BMI1^{High};CHD7^{Low} vs CTRL) for direct targets upregulated (UP) or downregulated (DOWN) upon PTC treatment (right).

C-D. Venn diagrams representing BMI1/MAPK shared targets in CTRL (C) and BMI1^{High};CHD7^{Low} (D) (top). Histograms showing LogFC values of BMI1/MAPK shared targets obtained from RNA-Seq analysis upon PTC (BMI1-responding genes, dark purple) or PD (MAPK-responding genes, dark blue) treatment.

E. Venn diagram showing overlaps between genes involved in MAPK signalling (black) and genes with promoter bound by BMI1 in CTRL (green) or BMI1^{High};CHD7^{Low} (red). CTRL-specific genes names are reported.

F. Matrices reporting Loewe Synergy Scores obtained after combination treatment of PTC and PD in CTRL or BMI1^{High};CHD7^{Low} CHLA-01-Med MB cells. n=3 biological independent experiments.

G. Violin plot showing Log10CI (CI reported in Figure 3G) for CTRL or BMI1^{High};CHD7^{Low} (B^{High};C^{Low}) CHLA-01-Med MB cells treated with the indicated combination of PTC (different symbols) and PD (different shades of colours). n=3 independent biological experiments, unpaired t-test.

All graphs report mean ± SEM.

Figure S4 Badodi et al

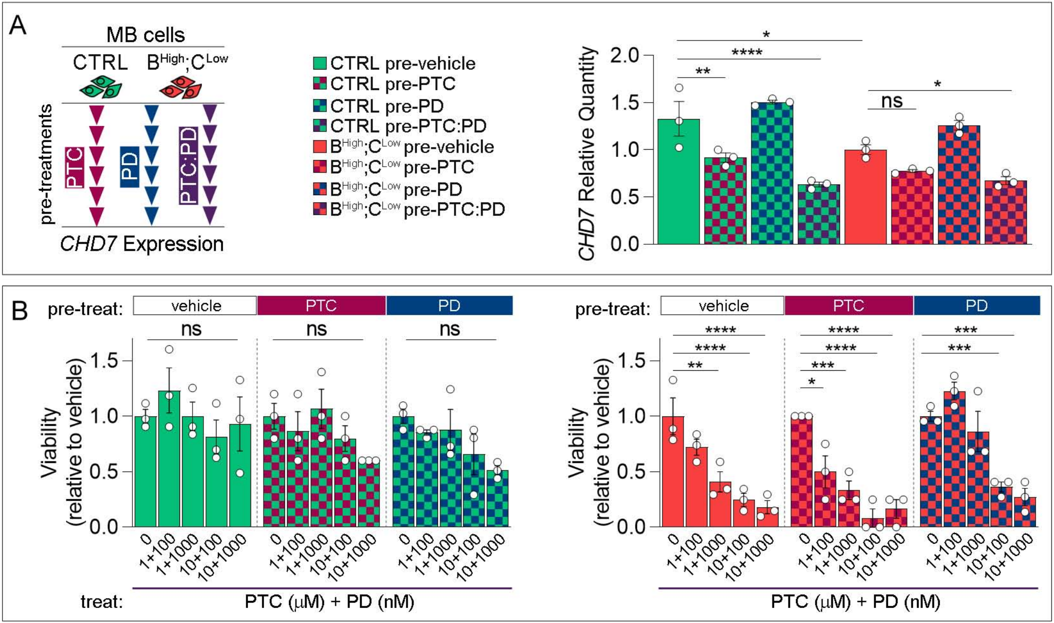

Fig. S4. PTC:PD treatment prevents acquired therapy resistance.

A. RT-qPCR analysis showing quantification of *CHD7* expression in control (CTRL, green) and BMI1^{High};CHD7^{Low} (B^{High};C^{Low},red) CHLA-01-Med MB cells upon PTC, PD or PTC:PD pre-treatments as in Figure 4. n=3 biological independent experiments, one-way ANOVA.

B. Cell viability assays of CTRL (D) or BMI1^{High};CHD7^{Low} (E) CHLA-01-Med MB cells pre-treated with PTC, PD or PTC:PD as previously and upon combined treatment with PTC-209 and PD325901 (PTC:PD). n=3 independent biological experiments, two-way ANOVA.

All graphs report mean ± SEM.

Figure S5

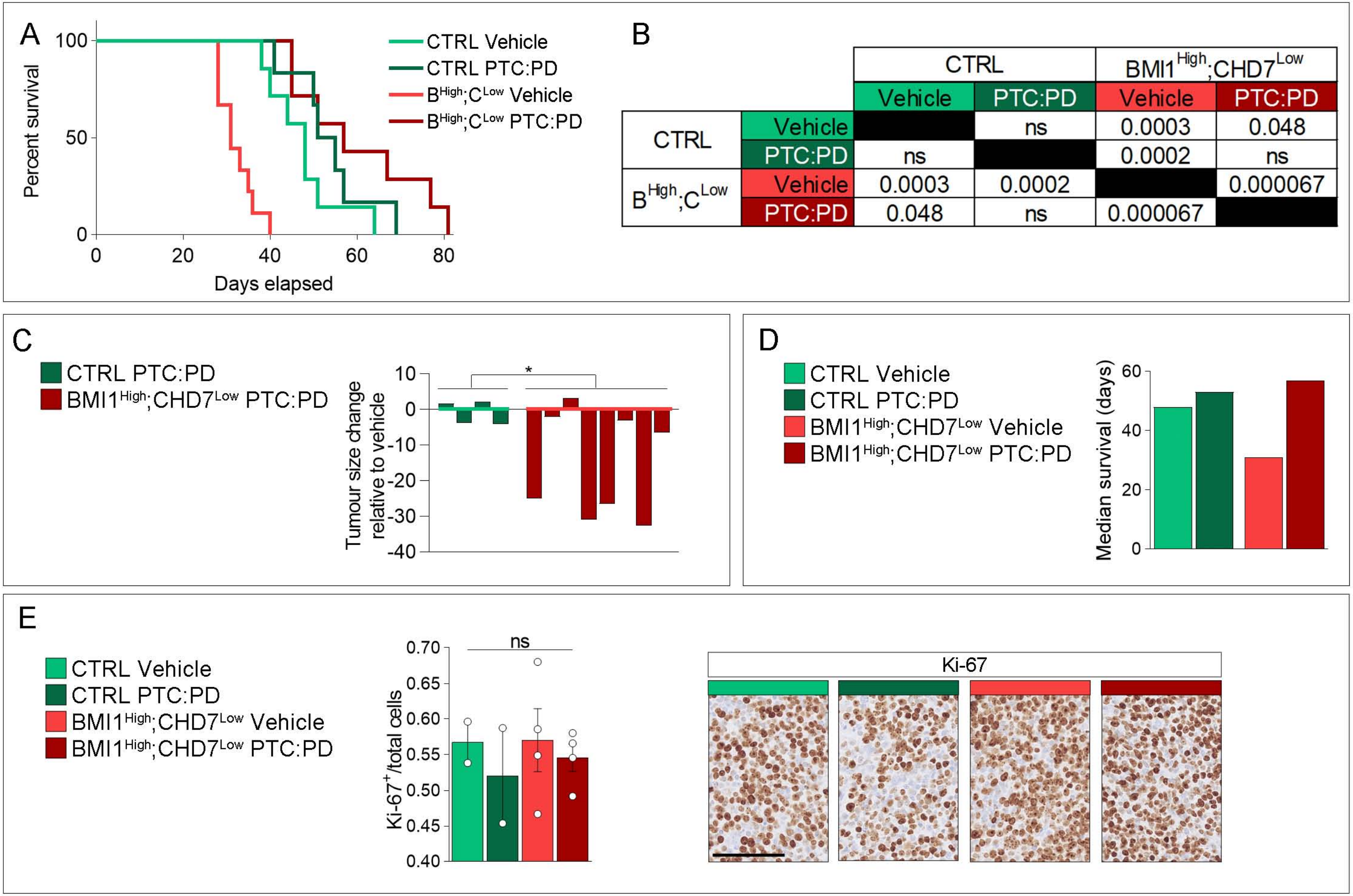
A
Percent survival
Days elapsed
100
50
0
0
20
40
60
80
CTRL Vehicle
CTRL PTC:PD
B^High;C^Low Vehicle
B^High;C^Low PTC:PD
B
CTRL
BMI1^High;CHD7^Low
Vehicle
PTC:PD
Vehicle
PTC:PD
CTRL Vehicle: —, ns, 0.0003, 0.048
CTRL PTC:PD: ns, —, 0.0002, ns
B^High;C^Low Vehicle: 0.0003, 0.0002, —, 0.000067
B^High;C^Low PTC:PD: 0.048, ns, 0.000067, —
C
CTRL PTC:PD
BMI1^High;CHD7^Low PTC:PD
Tumour size change relative to vehicle
*
10
0
-10
-20
-30
-40
D
CTRL Vehicle
CTRL PTC:PD
BMI1^High;CHD7^Low Vehicle
BMI1^High;CHD7^Low PTC:PD
Median survival (days)
60
40
20
0
E
CTRL Vehicle
CTRL PTC:PD
BMI1^High;CHD7^Low Vehicle
BMI1^High;CHD7^Low PTC:PD
Ki-67+/total cells
ns
0.70
0.65
0.60
0.55
0.50
0.45
0.40
Ki-67

Fig. S5. PTC:PD treatment extends survival of BMI1^{High};CHD7^{Low} MB xenografts.

A. Kaplan-Meier survival curves of mice orthotopically xenografted with control (CTRL, green) or BMI1^{High};CHD7^{Low} (B^{High};C^{Low}, red) CHLA-01-Med MB cells treated with vehicle or PTC:PD. n=7 biological independent animal per CTRL vehicle, 6 per CTRL PTC:PD, 9 per BMI1^{High};CHD7^{Low} vehicle and 7 per BMI1^{High};CHD7^{Low} PTC:PD.

B. Table reporting two-tailed p-values of Kaplan-Meier survival curves in Figure S5A determined by log-rank test.

C. Histograms showing changes in the area of the tumours developed in mice treated with PTC:PD compared to vehicle, unpaired t-test.

D. Histograms showing median survival day of mice treated as in A.

E. Quantification of fractions of ki-67 positive cells of total number of tumour cells in mice treated as described in A. n=2 per CTRL groups and n=4 per BMI1^{High};CHD7^{Low} groups, one-way ANOVA.

All graphs report mean ± SEM. Scale bars=100μm.

Figure S6

Badodi et al

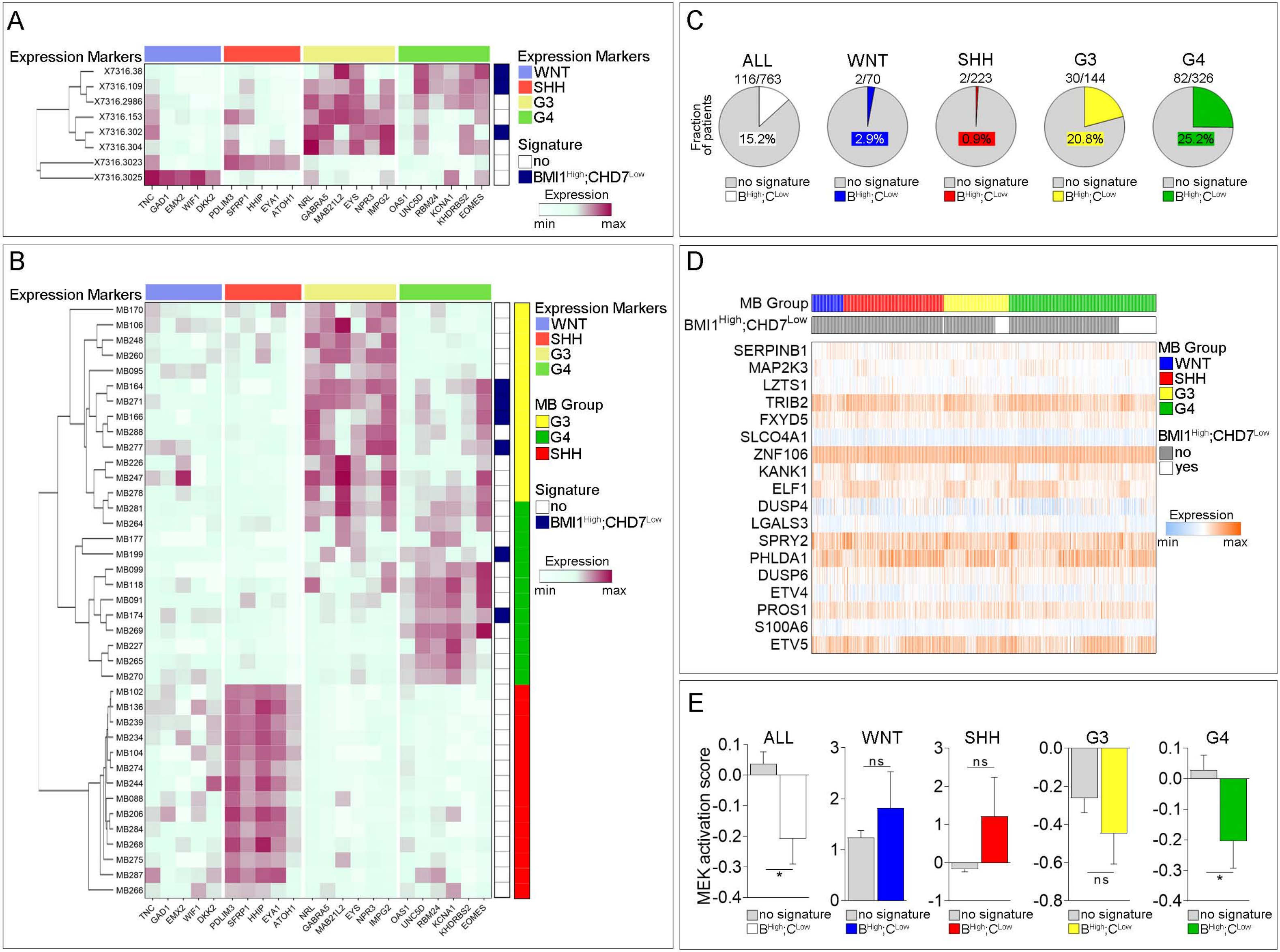

Fig. S6. Correlation between BMI1^{High};CHD7^{Low} and MAPK/ERK pathway activity is predicted by phospho-proteomic but not transcriptomic analysis.

A. Heatmap showing expression of marker genes associated with different MB subgroups in samples represented in Figure 6C, D. Presence of BMI1^{High};CHD7^{Low} signature is reported.

B. Heatmap showing expression of marker genes which allows clustering of MB patients (shown in Figure 6G) based on MB subgroup. Previously published subgroup affiliation for the analysed samples[23] is reported next to the heatmap and confirms classification based on expression markers. Presence of BMI1^{High};CHD7^{Low} signature is reported.

C. Pie charts showing percentages of BMI1^{High};CHD7^{Low} patients within each MB subgroups in the cohort analysed in Figure S5D,E.

D. Heatmap showing expression of the 18 genes included in the MEK activation signature in MB patients. MB subgroup affiliation and presence of BMI1^{High};CHD7^{Low} signature are reported.

E. Histogram showing ssGSEA MEK activation scores in MB patients with or without BMI1^{High};CHD7^{Low} signature from all or each MB subgroups. ALL: n=116 per BMI1^{High};CHD7^{Low} and n=647 per no signature, WNT: n=2 per BMI1^{High};CHD7^{Low} and n=68 per no signature, SHH: n=2 per BMI1^{High};CHD7^{Low} and n=221 per no signature, G3: n=30 per BMI1^{High};CHD7^{Low} and n=114 per no signature, G4: n=82 per BMI1^{High};CHD7^{Low} and n=244 per no signature, unpaired t-test.

All graphs report mean ± SEM.
